# Performance Analysis of Relay-Aided Hybrid FSO/RF Cooperation Communication System over the Generalized Turbulence Channels with Pointing Errors and Nakagami-m Fading Channels

**DOI:** 10.3390/s23136191

**Published:** 2023-07-06

**Authors:** Yan Wu, Gang Li, Dejin Kong

**Affiliations:** The State Key Laboratory of New Textile Materials and Advanced Processing Technologies, School of Electronic and Electrical Engineering, Wuhan Textile University, Wuhan 430200, China; wuyan@wtu.edu.cn (Y.W.); gli@wtu.edu.cn (G.L.)

**Keywords:** relay-aided hybrid FSO/RF system, atmospheric turbulence, Nakagami fading, pointing errors

## Abstract

To improve the performance of fee-space optical communication systems, this paper analyzes the performance of a relay-aided hybrid fee-space optical (FSO)/radio frequency (RF) cooperation system based on a selective combination and decoding forward transmission scheme. In this system, the FSO sub-link experienced Málaga turbulence with pointing errors and the RF sub-link suffered Nakagami-m fading. Firstly, the probability density function (PDF) and cumulative distribution function (CDF) of the end-to-end output signal-to-noise ratio (SNR) of the relay-aided hybrid FSO/RF system are derived. Then, using the extended generalized bivariate Meijer’s G-function (EGBMGF) and the approximate analytical formula of the generalized Gauss–Laguerre integral, mathematical expressions of the end-to-end average bit error rate (ABER) and outage probability of the relay-aided hybrid FSO/RF system with different subcarrier intensity modulation and different detection schemes are derived. Through a simulation analysis of the system, the results show that compared with the other three modulation technologies, the hybrid FSO/RF direct link and relay-aided hybrid FSO/RF system with coherent binary phase shift keying (CBPSK) modulation have the best bit error performance. Compared with direct detection, the hybrid direct link and relay-aided hybrid system with coherent detection can significantly improve the communication performance. Increasing the RF fading parameter *m* can further improve the bit error and outage performance of the hybrid direct link and relay-aided hybrid system; the hybrid direct link can significantly mitigate the degradation of communication performance in the FSO system caused by pointing errors, and the relay-aided hybrid system can further improve the communication performance; under weak turbulence conditions, the impact of pointing errors on the performance of the relay-aided hybrid system can even be ignored. The greater the total number of paths in the relay-aided hybrid system, the better the communication performance of the system; however, the more hops, the worse the performance of the system. The outage probability of the hybrid direct link and relay-aided hybrid system are very sensitive to the decision threshold, and the larger the decision threshold, the worse the outage performance. The transmission distance of different hybrid direct links has little impact on the performance of hybrid direct links and relay-aided hybrid systems. Improving the signal-to-noise ratio of RF sub-links significantly improves the performance of hybrid direct links and relay-aided hybrid systems.

## 1. Introduction

In recent years, free-space optical (FSO) communication systems have combined the advantages of microwave communication and optical fiber communication. They have the characteristics of large transmission capacity, fast network construction, being license-free, good security, and so on. They are usually used as the “last-kilometer” of telecommunications or LAN links between buildings [1]. However, the performance of terrestrial FSO communication systems is not only affected by atmospheric channels, such as the weather (cloud, fog, rain, snow, haze, etc.) and optical turbulence, but also affected by the pointing errors [2,3].

In order to mitigate the impact of the atmospheric environment on the performance of terrestrial FSO communication systems and improve the reliability and availability of terrestrial FSO communication systems, a hybrid FSO/radio frequency (RF) transmission system combining an FSO link and a millimeter-wave RF link was proposed [4]. Usman et al. [5] proposed a hybrid FSO/RF transmission scheme based on hard switching (only one link transmits information at the same time) and derived the expressions of the outage probability, average bit error rate (ABER), and ergodic channel capacity (ECC) of the system. Shrivastava et al. [6] proposed a hybrid FSO/RF system with a switching scheme according to the channel state information (CSI), and deduced the closed-form expressions of the ABER and outage probability under a strong turbulent environment. Shakir [7] derived new closed-form expressions of the BER and outage probability of a hybrid FSO/RF parallel communication system based on selective combination technology. The results showed that the performance of the hybrid FSO/RF parallel system was better than that of only the FSO system or only the RF system under different atmospheric environments.

In order to improve the transmission distance and coverage of terrestrial FSO communication systems, a multi-hop network based on FSO communication was proposed [8]. Zedini et al. [9] analyzed the performance of the multi-hop FSO system based on amplify-and-forward (AF) transmission and the channel state information (CSI) assisted or fixed gain relay, and derived new closed-form expressions for the end-to-end ABER and ECC of the system under different binary modulation schemes. Wang et al. [10] proposed a multi-hop FSO communication system based on decode-and-forward (DF) transmission and orthogonal frequency division multiplexing (OFDM), and derived closed expressions of the outage probability and ABER of the system.

In order to further improve the transmission performance of multi-hop networks, a parallel multi-hop cooperative communication network was proposed, that is, a relay-aided system [11]. Gao et al. [12] deduced the mathematical expressions of the ABER and outage probability of the relay-aided multi-hop network based on binary phase shift keying (BPSK) modulation under Gamma–Gamma turbulence with pointing errors. Wang et al. [13] deduced the end-to-end ABER and outage probability of the multi-hop parallel FSO system under the Málaga turbulent channel with pointing errors and a DF relay scheme, and analyzed the performance of the system under different turbulent intensity, weather conditions, pointing errors, and relay-aided structures. Androutsos et al. [14] minimized the mathematical complexity in analyzing outage and error performance by emulating a multi-hop terrestrial FSO communication system with various modulation formats as a single dual-hop scheme. Mohammad et al. [15,16] proposed a multi-user multi-hop hybrid FSO/RF communication system, and deduced and analyzed the mathematical expressions of the system performance.

Thus, motivated by the above-mentioned facts and considering its military and disaster recovery applications, we first proposed a new communication scheme combining the hybrid FSO/RF parallel transmission and the multi-hop parallel relay-aided communication in Ref. [17], that is, the multi-hop parallel hybrid FSO/RF cooperation system or the relay-aided hybrid FSO/RF system. Compared with previous work, the innovative content of this article includes the following points:

- First, we used the gamma–gamma turbulence model in our previous work, while we used the more general Málaga turbulence model in this paper, resulting in a new performance expression.
- Second, due to model limitations, we only derived and analyzed the system performance under CBPSK and DBPSK modulation methods in the previous work. In this paper, we derived and analyzed the system performance under four modulation methods (CBPSK, DBPSK, CBFSK, NBFSK).
- Third, we only derived and analyzed the system performance under direct detection in the previous work. In this paper, we derived and compared the system performance under direct detection and coherent detection.
- Finally, we derived and analyzed the relationship between the system outage probability and signal-to-noise ratio in the previous work. In this paper, we derived and analyzed the relationship between the system outage probability and the decision threshold.

The remainder of this paper, considers direct detection (DD) and heterodyne detection (HD), the PDF and CDF of the output SNR of only the FSO direct link and only the RF direct link are given, respectively. Furthermore, the CDF of the output SNR of the hybrid FSO/RF parallel link in the one-hop or direct link is obtained. Then, the end-to-end performance of the multi-hop parallel hybrid FSO/RF cooperative system based on the DF protocol is analyzed, and new mathematical expressions of the end-to-end ABER and outage probability of the system under four binary subcarrier modulation schemes are derived. Finally, through numerical analysis, the effects of the turbulence, pointing errors, modulation mode, detection technique, RF fading, and relay-aided structure on the hybrid FSO/RF direct link and the relay-aided hybrid system are compared and analyzed.

## 2. System and Channel Model

**Figure 1:**
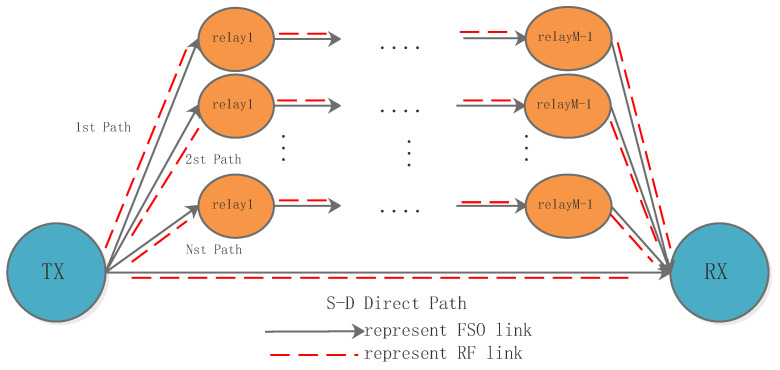
Schematic of the multi-hop parallel hybrid FSO/RF cooperative communication system.

Figure 1 shows a schematic of the multi-hop parallel hybrid FSO/RF cooperative communication system. On each branch of the relay-aided hybrid system, the signal is separated into two identical signals after subcarrier modulation, and the signals are transmitted through the FSO and RF transmitter, respectively. According to the principle of maximum SNR, any regenerative repeater will select the maximum SNR signal from the two received signals for demodulation, regeneration, and forwarding.

For the relay-aided hybrid system with an (N,M) structure, the system has *N* cooperation paths and one direct path. For any cooperation path, there are M-hops from the source node to the destination node. Only one relay is allowed to process the received signals at one time based on the symbol-wise DF relaying method. Based on the best path selection scheme, a cooperative path is chosen to implement the transmission of the digital signal from the source node to the destination node.

### 2.1. One-Hop FSO Sub-Link

The Málaga distribution has unified most of the existing turbulence models, such as gamma–gamma distribution, log-normal distribution, κ distribution, and so on [18]. Considering the Málaga turbulence with pointing errors, the PDF of the output instantaneous SNR $\gamma_{}^{\mathrm{FSO}}$ of the one-hop FSO sub-link is [19]:

(1) $$f_{\gamma_{}^{\mathrm{FSO}}}\left(\gamma_{}^{\mathrm{FSO}}\right)=\frac{\xi_{}^{2}A}{2^{r}\gamma_{}^{\mathrm{FSO}}}\sum_{k=1}^{\beta }b_{k}G_{1,3}^{3,0}\left(B{\left(\frac{\gamma_{}^{\mathrm{FSO}}}{\mu_{}^{\mathrm{FSO},r}}\right)}^{\frac{1}{r}}\left|\begin{array}{c} 1+\xi_{}^{2}\\ \xi_{}^{2},\alpha ,k \end{array}\right.\right),$$

where $G_{\cdot ,\cdot }^{\cdot ,\cdot }\left(\cdot \right)$ is the Meijer-G function, ξ is the ratio of the equivalent beam radius of the receiver plane to the jitter standard deviation, $B=\xi^{2}\alpha \beta \left(g\beta +\Omega^{\prime }\right)/\left[\left(\xi^{2}+1\right)\left(g\beta +\Omega^{\prime }\right)\right]$, and $b_{k}=a_{k}{\left[\alpha \beta /(g\beta +\Omega^{\prime })\right]}^{-\left(\alpha +k\right)/2}$,

(2) $$\left\{\begin{array}{c} A=\frac{2\alpha^{\alpha /2}}{g^{1+\alpha /2}\Gamma \left(\alpha \right)}{\left(\frac{g\beta }{g\beta +\Omega }\right)}^{\beta +\frac{\alpha }{2}}\\ a_{k}=\left(\begin{array}{c} \beta -1\\ k-1 \end{array}\right)\frac{{\left(g\beta +\Omega \right)}^{1-\frac{k}{2}}}{(k-1)!}{\left(\frac{\Omega }{g}\right)}^{k-1}{\left(\frac{\alpha }{\beta }\right)}^{k/2} \end{array}\right.,$$

where α is a positive parameter, β is a natural number, Γ(·) is the Gamma function. $g=2b_{0}\left(1-\rho \right)$, $2b_{0}$ represents the average power of the total scattering component, ρ (0≤ρ≤1) represents the scattering power coupled with the line-of-sight component, Ω represents the average power of mutual coupling components, $\mu_{}^{\mathrm{FSO},r}$ represents the average SNR of the FSO sub-link, and *r* is the parameter that defines the detection technology, that is, r=1 represents HD and r=2 represents DD. When r=1,

(3) $$\mu_{}^{\mathrm{FSO},1}=\mu_{\mathrm{heterodyne}}={\bar{\gamma }}^{\mathrm{FSO}};$$

when r=2,

(4) $$\mu_{}^{\mathrm{FSO},2}=\mu_{\mathrm{IM}/\mathrm{DD}}=\frac{\alpha \xi^{2}{\left(\xi^{2}+1\right)}^{-2}\left(\xi^{2}+2\right)\left(g+\Omega^{\prime }\right)}{\left(\alpha +1\right)\left[2g\left(g+2\Omega^{\prime }\right)+\Omega {}^{\prime 2}\left(1+\frac{1}{\beta }\right)\right]}\mu_{}^{\mathrm{FSO},1}.$$

Integrate Equation (1) with the formula $F_{\gamma }\left(\gamma \right)=\int_{0}^{\gamma }f_{\gamma }\left(t\right)dt$, and the CDF of $\gamma_{}^{\mathrm{FSO}}$ can be expressed as [19]:

(5) $$F_{\gamma_{}^{\mathrm{FSO}}}\left(\gamma_{}^{\mathrm{FSO}}\right)=D\sum_{k=1}^{\beta }c^{k}G_{r+1,3r+1}^{3r,1}\left(E\frac{\gamma_{}^{\mathrm{FSO}}}{\mu^{\mathrm{FSO},r}}\left|\begin{array}{c} 1,\kappa^{1}\\ \kappa^{2},0 \end{array}\right.\right),$$

where $D=\xi^{2}A/\left[2^{r}{\left(2\pi \right)}^{r-1}\right]$, $c^{k}=b_{k}r^{\alpha +k-1}$, $E=B^{r}/r^{2r}$, $\kappa^{1}=\left(\xi^{2}+1\right)/r,\ldots ,\left(\xi^{2}+r\right)/r$ (include *r* items), and $\kappa^{2}=\xi^{2}/r,\ldots ,\left(\xi^{2}+r-1\right)/r,\alpha /r,\ldots ,\left(\alpha +r-1\right)/r,k/r,\ldots ,\left(k+r-1\right)/r$ (include 3r items).

### 2.2. One-Hop RF Sub-Link

In the one-hop RF sub-link with Nakagami-m fading channel, the PDF of the SNR $\gamma_{}^{\mathrm{RF}}$ is [20]:

(6) $$f_{\gamma_{}^{\mathrm{RF}}}\left(\gamma_{}^{\mathrm{RF}}\right)={\left(\frac{m}{{\bar{\gamma }}_{}^{\mathrm{RF}}}\right)}^{m}\frac{\gamma^{m-1}}{\Gamma (m)}\exp\left(-\frac{m\gamma_{}^{\mathrm{RF}}}{{\bar{\gamma }}_{}^{\mathrm{RF}}}\right)={\left(\frac{m}{{\bar{\gamma }}_{}^{\mathrm{RF}}}\right)}^{m}\frac{\gamma^{m-1}}{\Gamma (m)}G_{0,1}^{1,0}\left[\frac{m\gamma_{}^{\mathrm{RF}}}{{\bar{\gamma }}_{}^{\mathrm{RF}}}\left|\begin{array}{c} -\\ 0 \end{array}\right.\right],$$

where ${\bar{\gamma }}_{}^{\mathrm{RF}}$ is the average SNR of the one-hop RF sub-link, and *m* (m≥0.5) is the fading parameter of the one-hop RF sub-link. The CDF of the SNR $\gamma_{}^{\mathrm{RF}}$ can be obtained by integration,

(7) $$F_{\gamma_{}^{\mathrm{RF}}}\left(\gamma_{}^{\mathrm{RF}}\right)=\frac{1}{\Gamma (m)}G_{1,2}^{1,1}\left(\frac{m\gamma_{}^{\mathrm{RF}}}{{\bar{\gamma }}_{}^{\mathrm{RF}}}\left|\begin{array}{c} 1\\ m,0 \end{array}\right.\right).$$

### 2.3. One-Hop Hybrid FSO/RF Link Based on Selective Combination Scheme

In the one-hop hybrid FSO/RF sub-link with the selective combination scheme, the receiver detects two branch signals at the same time and selects the signal with the maximum SNR to demodulate. Then, the output SNR $\gamma_{}^{\mathrm{SC}}$ of the selective combiner on the one-hop hybrid link can be expressed as [15]:

(8) $$\gamma_{}^{\mathrm{SC}}=\max\left(\gamma_{}^{\mathrm{FSO}},\gamma_{}^{\mathrm{RF}}\right).$$

The CDF of the SNR $\gamma_{}^{\mathrm{SC}}$ can be expressed as [15]:

(9) $$F_{\gamma_{}^{\mathrm{SC}}}\left(\gamma \right)=\mathrm{Pr}(\max(\gamma_{}^{\mathrm{FSO}},\gamma_{}^{\mathrm{RF}})\leq \gamma )=\mathrm{Pr}(\gamma_{}^{\mathrm{FSO}}\leq \gamma ,\gamma_{}^{\mathrm{RF}}\leq \gamma )=F_{\gamma_{}^{\mathrm{FSO}}}\left(\gamma \right)F_{\gamma^{\mathrm{RF}}}\left(\gamma \right).$$

Adding subscripts (*x*, *y*) to all variables in the Málaga turbulence model and Nakagami fading channel, when the subscript is (*x*, *y*) = (*i*, *j*), it represents the *j*-th hop hybrid FSO/RF link of the *i*-th path, when the subscript is (*x*, *y*) = (*s*, *d*), it represents the direct hybrid FSO/RF link. Substituting Equations (5) and (7) into Equation (9), the CDF of the selective combiner output SNR $\gamma_{s,d}^{\mathrm{SC}}$ of the direct channel and the CDF of the selective combiner output SNR $\gamma_{i,j}^{\mathrm{SC}}$ of the *j*-th hop of the *i*-th path can be obtained as follows:

(10) $$F_{\gamma_{x,y}^{\mathrm{SC}}}\left(\gamma \right)=\frac{D_{x,y}}{\Gamma (m_{x,y})}\sum_{k=1}^{\beta_{x,y}}c_{x,y}^{k}G_{1,2}^{1,1}\left(\frac{m_{x,y}\gamma }{{\bar{\gamma }}_{x,y}^{\mathrm{RF}}}\left|\begin{array}{c} 1\\ m_{x,y},0 \end{array}\right.\right)G_{r+1,3r+1}^{3r,1}\left(E_{x,y}\frac{\gamma }{\mu_{x,y}^{\mathrm{FSO},r}}\left|\begin{array}{c} 1,\kappa_{x,y}^{1}\\ \kappa_{x,y}^{2},0 \end{array}\right.\right).$$

According to formula (07.34.16.0003.01) in Ref. [21], the product of two Meijer-G functions in Equation (10) can be replaced by the extended generalized bivariate Meijer’s G-function (EGBMGF),

(11) $$\;F_{\gamma_{x,y}^{\mathrm{SC}}}\left(\gamma \right)\;=\;\frac{D_{x,y}}{\Gamma (m_{x,y})}\left[\sum_{k=1}^{\beta_{x,y}}c_{x,y}^{k}G_{0,0;1,2;r+1,3r+1}^{0,0;1,1;3r,1}\;\left(\;\left.\;\begin{array}{c} \;\;-\;\;\\ \;\;-\;\; \end{array}\right|\right.\;\left.\;\begin{array}{c} 1\\ m_{x,y},\;0 \end{array}\;\right|\;\left.\;\begin{array}{c} 1,\;\kappa_{x,y}^{1}\\ \kappa_{x,y}^{2},\;0 \end{array}\;\right|\;\left.\;\frac{m_{x,y}\gamma }{{\bar{\gamma }}_{x,y}^{\mathrm{RF}}},\frac{E_{x,y}\gamma }{\mu_{x,y}^{\mathrm{FSO},r}}\;\right)\;\right]\;.$$

## 3. End-to-End System Performance Analysis

For the relay-aided hybrid FSO/RF cooperative system with DF protocol, the equivalent SNR $\gamma_{{\mathrm{eq}}_{i}}$ of the *i*-th path can be expressed as [13]:

(12) $$\gamma_{{\mathrm{eq}}_{i}}=\min\left(\gamma_{i,1}^{\mathrm{SC}},\gamma_{i,2}^{\mathrm{SC}},\cdots ,\gamma_{i,M}^{\mathrm{SC}}\right).$$

Considering that each branch of the relay-aided hybrid FSO/RF system is independent and identically distributed, the CDF of the equivalent SNR $\gamma_{{\mathrm{eq}}_{i}}$ of the *i*-th path can be expressed as [13]:

(13) $$F_{\gamma_{{\mathrm{eq}}_{i}}}\left(\gamma \right)=1-{\left[1-F_{\gamma_{i,j}^{\mathrm{SC}}}\left(\gamma \right)\right]}^{M}.$$

Based on the best path selection scheme, the receiver of the destination node selects the highest SNR signal from the *i*-th path (the SNR $\gamma_{{\mathrm{eq}}_{i}}$, i=1,…,N) and the direct path (the SNR $\gamma_{s,d}^{\mathrm{SC}}$) signals for output. Therefore, the equivalent output SNR $\gamma_{\mathrm{eq}}$ at the destination node can be expressed as:

(14) $$\gamma_{\mathrm{eq}}\;=\max\left(\gamma_{s,d}^{\mathrm{SC}},\gamma_{\mathrm{eq}}^{\prime }\right),$$

where $\gamma_{\mathrm{eq}}^{\prime }=\max_{i=1,\ldots N}\gamma_{{\mathrm{eq}}_{i}}$, and the CDF of $\gamma_{\mathrm{eq}}^{\prime }$ can be written as:

(15) $$\begin{array}{cc} \;F_{{\gamma^{\prime }}_{{\mathrm{eq}}_{i}}}\left(\gamma \right)\; & =\;\left[\;F_{\gamma_{{\mathrm{eq}}_{i}}}\left(\gamma \right)\;\right]{\;}^{N}\;=\;{\left[\;1-\;{\left[\;1\;-\;F_{\gamma_{i,j}^{\mathrm{SC}}}\left(\gamma \right)\right]}^{M}\right]}^{N}\\ \; & =\;{\left\{\;1\;-\;{\left\{\;1\;-\;\frac{D_{i,j}}{\Gamma (m_{i,j})}\left[\;\sum_{k=1}^{\beta_{i,j}}c_{i,j}^{k}G_{0,0;1,2;r+1,3r+1}^{0,0;1,1;3r,1}\;\left(\;\left.\begin{array}{c} \;\;-\;\;\\ \;\;-\;\; \end{array}\right|\right.\;\left.\;\begin{array}{c} 1\;\\ m_{i,j},\;0\;\; \end{array}\;\right|\;\left.\;\begin{array}{c} 1,\;\kappa_{i,j}^{1}\;\;\\ \kappa_{i,j}^{2},\;0\;\; \end{array}\;\right|\;\left.\;\frac{m_{i,j}\gamma }{{\bar{\gamma }}_{i,j}^{\mathrm{RF}}},\;\frac{E_{i,j}\gamma }{\mu_{i,j}^{\mathrm{FSO},r}}\right)\;\right]\;\right\}}^{M}\;\right\}}^{N}\;. \end{array}$$

Then, the CDF of the output SNR of the destination node is

(16) $$\begin{array}{cc} \;F_{\gamma_{\mathrm{eq}}}\left(\gamma \right)\; & =\;F_{\gamma_{s,d}^{\mathrm{SC}}}\left(\gamma \right)\;\times \;F_{{\gamma^{\prime }}_{{\mathrm{eq}}_{i}}}\left(\gamma \right)\;=\;F_{\gamma_{s,d}^{\mathrm{SC}}}\left(\gamma \right)\times {\left[1\;-\;{\left[1\;-\;F_{\gamma_{i,j}^{\mathrm{SC}}}\left(\gamma \right)\right]}^{M}\right]}^{N}\\ \; & =\;\frac{D_{s,d}}{\Gamma (m_{s,d})}\left[\sum_{k=1}^{\beta_{s,d}}c_{s,d}^{k}G_{0,0;1,2;r+1,3r+1}^{0,0;1,1;3r,1}\;\left(\;\left.\;\begin{array}{c} \;\;-\;\\ \;\;-\; \end{array}\right|\right.\left.\begin{array}{c} 1\\ m_{s,d},\;0 \end{array}\;\right|\left.\;\begin{array}{c} 1,\;\kappa_{s,d}^{1}\\ \kappa_{s,d}^{2},\;0 \end{array}\;\right|\left.\;\frac{m_{s,d}\gamma }{{\bar{\gamma }}_{s,d}^{\mathrm{RF}}},\frac{E_{s,d}\gamma }{\mu_{s,d}^{\mathrm{FSO},r}}\;\right)\;\right]\;\\ \; & \times \;{\left\{\;1-{\left\{1\;-\;\frac{D_{i,j}}{\Gamma (m_{i,j})}\left[\;\sum_{k=1}^{\beta_{i,j}}c_{i,j}^{k}G_{0,0;1,2;r+1,3r+1}^{0,0;1,1;3r,1}\;\left(\;\left.\;\begin{array}{c} \;\;-\;\\ \;\;-\; \end{array}\right|\right.\left.\begin{array}{c} 1\\ m_{i,j},\;0 \end{array}\;\right|\;\left.\;\begin{array}{c} 1,\;\kappa_{i,j}^{1}\\ \kappa_{i,j}^{2},\;0 \end{array}\;\right|\;\left.\;\frac{m_{i,j}\gamma }{{\bar{\gamma }}_{i,j}^{\mathrm{RF}}},\frac{E_{i,j}\gamma }{\mu_{i,j}^{\mathrm{FSO},r}}\;\right)\;\right]\;\right\}}^{M}\;\right\}}^{N}\;. \end{array}$$

### 3.1. Average Bit Error Rate

For the relay-aided hybrid FSO/RF cooperation system, four binary modulation schemes are used for data transmission in any FSO or RF sub-link. According to Refs. [22,23], the expression of the ABER can be expressed as:

(17) $$P_{b}=\frac{q^{p}}{2\Gamma (p)}\int_{0}^{\infty }{\left(\gamma \right)}^{p-1}\exp\left(-q\gamma \right)F_{\gamma }\left(\gamma \right)d\gamma ,$$

where *p* and *q* are the ABER parameters used to describe four binary modulation schemes, as shown in Table 1.

By substituting Equation (16) into Equation (17), the ABER of the relay-aided hybrid system (*N*, *M*) can be obtained:

(18) $$\begin{array}{cc} P_{b}^{\mathrm{multi}}\; & =\;\frac{q^{p}}{2\Gamma (p)}\;\int_{0}^{\infty }\;{\left(\gamma \right)}^{p\;-\;1}\;\exp\;\left(-q\gamma \right)\;\frac{D_{s,d}}{\Gamma (m_{s,d})}\;\left[\;\sum_{k=1}^{\beta_{s,d}}c_{s,d}^{k}G_{0,0;1,2;r+1,3r+1}^{0,0;1,1;3r,1}\;\left(\;\left.\;\begin{array}{c} \;\;-\;\;\\ \;\;-\;\; \end{array}\right|\right.\;\left.\;\begin{array}{c} 1\\ m_{s,d},0 \end{array}\;\right|\;\left.\;\begin{array}{c} 1,\kappa_{s,d}^{1}\;\;\\ \kappa_{s,d}^{2},0\;\; \end{array}\;\right|\;\left.\;\frac{m_{s,d}\gamma }{{\bar{\gamma }}_{s,d}^{\mathrm{RF}}}\;,\frac{E_{s,d}\gamma }{\mu_{s,d}^{\mathrm{FSO},r}}\;\right)\;\right]\;\\ \; & \times \;{\left\{\;1\;-\;{\left\{\;1\;-\;\frac{D_{i,j}}{\Gamma (m_{i,j})}\;\left[\;\sum_{k=1}^{\beta_{i,j}}c_{i,j}^{k}G_{0,0;1,2;r+1,3r+1}^{0,0;1,1;3r,1}\;\left(\;\left.\begin{array}{c} \;\;-\;\;\\ \;\;-\;\; \end{array}\right|\right.\;\left.\;\begin{array}{c} 1\;\\ m_{i,j},\;0 \end{array}\;\right|\;\left.\;\begin{array}{c} 1,\;\kappa_{i,j}^{1}\;\\ \kappa_{i,j}^{2},\;0 \end{array}\;\right|\;\left.\;\frac{m_{i,j}\gamma }{{\bar{\gamma }}_{i,j}^{\mathrm{RF}}},\frac{E_{i,j}\gamma }{\mu_{i,j}^{\mathrm{FSO},r}}\;\right)\;\right]\;\right\}}^{M}\;\right\}}^{N}d\gamma . \end{array}$$

According to formula (3.6.1) in Refs. [24,25], $\;\int_{a}^{\infty }\;\;(x\;-\;a){\;}^{c}\;\exp\;\left(\;\;-b(x\;-\;a)\;\right)\;\;f\left(x\right)\;dx\;$ $\;\approx \;\sum_{\tau =1}^{n}w_{\tau }f\left(x_{\tau }\right)$ is the approximate expression of the generalized Gauss–Laguerre integral, where $w_{\tau }$ is the weight function and $x_{\tau }$ is the special point of abscissa. Therefore, Equation (18) can be simplified as:

(19) $$\begin{array}{cc} P_{b}^{\mathrm{multi}}\; & =\;\frac{q^{p}}{2\Gamma \;(p)}\frac{D_{s,d}}{\Gamma \;(m_{s,d})}\;\sum_{\tau =1}^{n}w_{\tau }\;\left[\;\sum_{k=1}^{\beta_{s,d}}c_{s,d}^{k}G_{0,0;1,2;r+1,3r+1}^{0,0;1,1;3r,1}\;\left(\;\left.\;\begin{array}{c} \;\;-\;\;\\ \;\;-\;\; \end{array}\right|\right.\;\left.\;\begin{array}{c} 1\;\\ m_{s,d},\;0 \end{array}\;\right|\;\left.\;\begin{array}{c} 1,\;\kappa_{s,d}^{1}\\ \kappa_{s,d}^{2},\;0 \end{array}\;\right|\;\left.\;\frac{m_{s,d}x_{\tau }}{{\bar{\gamma }}_{s,d}^{\mathrm{RF}}},\frac{E_{s,d}x_{\tau }}{\mu_{s,d}^{\mathrm{FSO},r}}\;\right)\;\right]\;\\ \; & \;\times \;{\left\{\;1\;-\;\;{\left\{\;1\;-\;\frac{D_{i,j}}{\Gamma (m_{i,j})}\;\left[\;\sum_{k=1}^{\beta_{i,j}}c_{i,j}^{k}G_{0,0;1,2;r+1,3r+1}^{0,0;1,1;3r,1}\;\left(\;\left.\;\begin{array}{c} \;\;-\;\;\\ \;\;-\;\; \end{array}\right|\right.\;\left.\;\begin{array}{c} 1\\ m_{i,j},\;0 \end{array}\;\right|\;\left.\;\begin{array}{c} 1,\;\kappa_{i,j}^{1}\\ \kappa_{i,j}^{2},\;0 \end{array}\;\right|\;\left.\;\frac{m_{i,j}x_{\tau }}{{\bar{\gamma }}_{i,j}^{\mathrm{RF}}},\frac{E_{i,j}x_{\tau }}{\mu_{i,j}^{\mathrm{FSO},r}}\;\right)\;\right]\;\right\}}^{M}\right\}}^{N}. \end{array}$$

According to Ref. [26] and q=1, $x_{\tau }$ is the τ-th root of the generalized Laguerre polynomial $L_{n}^{\left(-(1/2\right)}\left(x\right)$, and the corresponding weight coefficient is $w_{\tau }=\Gamma \left[n+(1/2)\right]x_{t}/$ $\left\{n!{\left(n+1\right)}^{2}{\left[L_{n+1}^{\left(-1/2\right)}\left(x_{t}\right)\right]}^{2}\right\}$.

According to formula (2.1) in Ref. [27], by substituting Equation (11) into Equation (17), the ABER of the direct link from the source node to the destination node can be obtained:

(20) $$\begin{array}{cc} \;P_{b}^{\mathrm{SD}}\; & =\;\frac{q^{p}D_{s,d}}{2\Gamma \left(p\right)\Gamma \left(m_{s,d}\right)}\;\sum_{k=1}^{\beta_{s,d}}c_{s,d}^{k}\;\int_{0}^{\infty }\;{\left(\gamma \right)}^{p\;-\;1}\exp\left(\;-\;q\gamma \right)G_{0,0;1,2;r+1,3r+1}^{0,0;1,1;3r,1}\;\left(\;\left.\;\begin{array}{c} \;\;-\;\;\\ \;\;-\;\; \end{array}\right|\;\right.\left.\;\begin{array}{c} 1\\ m_{s,d},\;0 \end{array}\;\right|\;\left.\;\begin{array}{c} 1,\kappa_{s,d}^{1}\\ \kappa_{s,d}^{2},0 \end{array}\;\right|\;\left.\;\frac{m_{s,d}\gamma }{{\bar{\gamma }}_{s,d}^{\mathrm{RF}}},\frac{E_{s,d}\gamma }{\mu_{s,d}^{\mathrm{FSO},r}}\;\right)\;d\gamma \\ \; & =\frac{D_{s,d}}{2\Gamma \left(p\right)\Gamma \left(m_{s,d}\right)}\sum_{k=1}^{\beta_{s,d}}c_{s,d}^{k}G_{0,0;1,2;r+1,3r+1}^{1,0;1,1;3r,1}\left(\left.\begin{array}{c} p\\ - \end{array}\right|\right.\left.\begin{array}{c} 1\\ m_{s,d},0 \end{array}\right|\left.\begin{array}{c} 1,\kappa_{s,d}^{1}\\ \kappa_{s,d}^{2},0 \end{array}\right|\left.\frac{m_{s,d}}{q{\bar{\gamma }}_{s,d}^{\mathrm{RF}}},\frac{E_{s,d}}{q\mu_{s,d}^{\mathrm{FSO},r}}\right). \end{array}$$

### 3.2. Outage Probability

The outage probability refers to the probability that the end-to-end output SNR is lower than a specific threshold $\gamma_{\mathrm{th}}$. Therefore, the outage probability of the system in this paper can be expressed as [13]:

(21) $$P_{\mathrm{out}}=\mathrm{Pr}\left(\gamma <\gamma_{\mathrm{th}}\right)=\int_{0}^{\gamma_{\mathrm{th}}}f_{\gamma }\left(\gamma \right)d\gamma =F_{\gamma }\left(\gamma_{\mathrm{th}}\right).$$

Substituting Equation (16) into Equation (21), the outage probability of the relay-aided hybrid FSO/RF cooperation system (*N*, *M*) can be obtained:

(22) $$\begin{array}{cc} P_{\mathrm{out}}^{\mathrm{multi}}\; & =\;\frac{D_{s,d}}{\Gamma (m_{s,d})}\;\left[\;\sum_{k=1}^{\beta_{s,d}}c_{s,d}^{k}G_{0,0;1,2;r+1,3r+1}^{0,0;1,1;3r,1}\;\left(\;\left.\;\begin{array}{c} \;\;-\;\;\\ \;\;-\;\; \end{array}\right|\right.\;\left.\;\begin{array}{c} 1\\ m_{s,d},0 \end{array}\;\right|\;\left.\;\begin{array}{c} 1,\kappa_{s,d}^{1}\\ \kappa_{s,d}^{2},\;0 \end{array}\;\right|\;\left.\frac{m_{s,d}\gamma_{\mathrm{th}}}{{\bar{\gamma }}_{s,d}^{\mathrm{RF}}},\frac{E_{s,d}\gamma_{\mathrm{th}}}{\mu_{s,d}^{\mathrm{FSO},r}}\right)\right]\\ \; & \;\times \;{\left\{\;1\;-\;{\;\left\{\;1\;-\;\frac{D_{i,j}}{\Gamma (m_{i,j})}\;\left[\sum_{k=1}^{\beta_{i,j}}c_{i,j}^{k}G_{0,0;1,2;r+1,3r+1}^{0,0;1,1;3r,1}\;\left(\;\left.\;\begin{array}{c} \;\;-\;\;\\ \;\;-\;\; \end{array}\right|\right.\;\left.\;\begin{array}{c} 1\\ m_{i,j},\;0 \end{array}\;\right|\;\left.\begin{array}{c} 1,\kappa_{i,j}^{1}\\ \kappa_{i,j}^{2},0 \end{array}\;\right|\;\left.\frac{m_{i,j}\gamma_{\mathrm{th}}}{{\bar{\gamma }}_{i,j}^{\mathrm{RF}}},\frac{E_{i,j}\gamma_{\mathrm{th}}}{\mu_{i,j}^{\mathrm{FSO},r}}\;\right)\;\right]\;\right\}}^{M}\right\}}^{N}. \end{array}$$

Substituting Equation (11) into Equation (21), the outage probability of the direct hybrid FSO/RF link can be obtained:

(23) $$P_{\mathrm{out}}^{\mathrm{SD}}=\frac{D_{s,d}}{\Gamma (m_{s,d})}\left[\sum_{k=1}^{\beta_{s,d}}c_{s,d}^{k}G_{0,0;1,2;r+1,3r+1}^{0,0;1,1;3r,1}\left(\left.\begin{array}{c} -\;\\ -\; \end{array}\;\right|\;\right.\;\left.\begin{array}{c} 1\\ m_{s,d},0 \end{array}\;\right|\;\left.\;\begin{array}{c} 1,\kappa_{s,d}^{1}\\ \kappa_{s,d}^{2},0 \end{array}\;\right|\;\left.\frac{m_{s,d}\gamma_{\mathrm{th}}}{{\bar{\gamma }}_{s,d}^{\mathrm{RF}}},\frac{E_{s,d}\gamma_{\mathrm{th}}}{\mu_{s,d}^{\mathrm{FSO},r}}\;\right)\;\right].$$

## 4. Numerical Results and Discussions

Under different turbulence conditions, pointing errors, RF fading parameters, modulation modes, detection schemes, and relay-aided structures, the performance of the hybrid FSO/RF direct link and the relay-aided hybrid system is numerically analyzed. By calculating the approximate value of the generalized Gauss–Laguerre quadrature function with τ=30, the ABER of the relay-aided hybrid system can be obtained according to Equation (19). To simplify the simulation analysis, it is assumed in this section that the atmospheric turbulence intensity is consistent for all FSO links in the relay-aided system (but the atmospheric scintillation index also varies depending on the transmission distance), and the fading parameters of all RF links are also the same. The structure parameters (N=2, M=3), (N=2, M=5), and (N=4, M=3) have been selected to avoid entanglement. According to the meaning of the scintillation index, the turbulence parameters set for the 1 km FSO link under the Málaga turbulence are selected as (α=5,β=3,ρ=0.25), (α=3,β=2,ρ=0.75), and (α=2,β=1,ρ=0.9), respectively, for weak, moderate, and strong turbulence conditions. For weak turbulence, the parameters of the Málaga turbulence are (α=0.36,β=1,ρ=0.95) and (α=0.0291,β=1,ρ=0.95), respectively, for 2 km and 3 km FSO links. Other parameters for the Málaga turbulence include Ω=1.3265, $b_{0}\;=\;0.1079$, and $\varphi_{A}-\varphi_{B}=\pi /2$.

**Figure 2:**
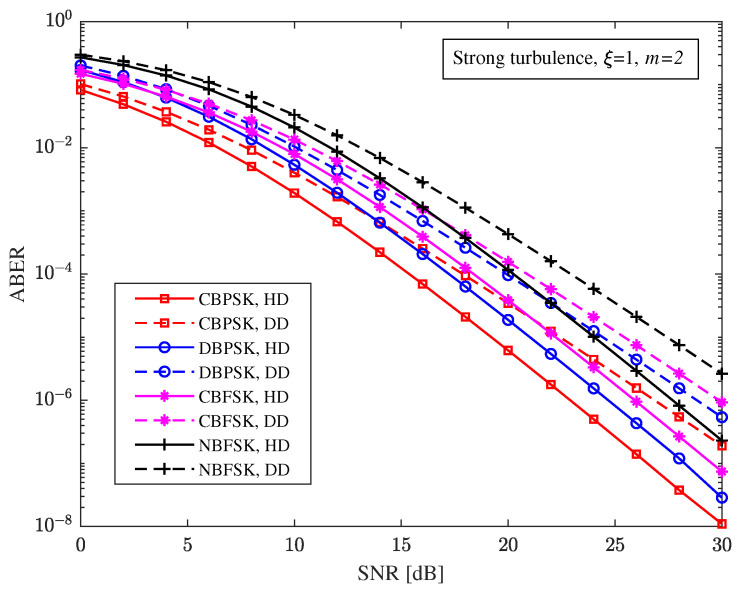
Relationship between ABER and SNR of hybrid FSO/RF direct link under different modulation modes and detection schemes.

**Figure 3:**
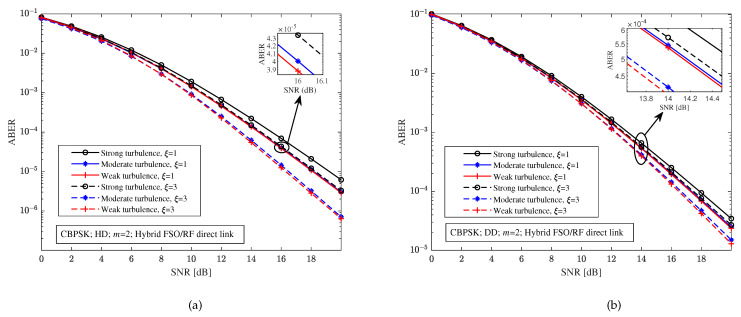
Relationship between the ABER and the SNR of hybrid FSO/RF direct link under different turbulence intensity, detection schemes, and pointing errors. (**a**) Heterodyne detection (HD). (**b**) Direct detection (DD).

**Figure 4:**
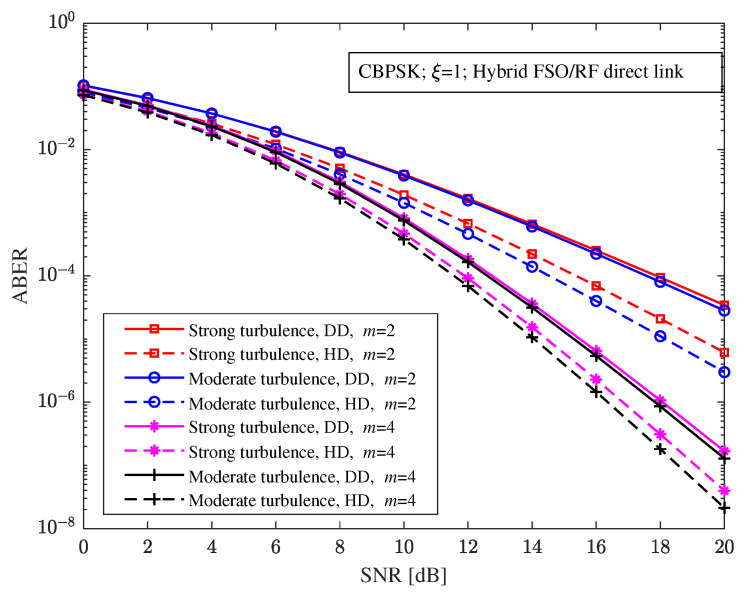
Relationship between the ABER and the SNR of the hybrid FSO/RF direct link under different turbulence intensity, detection schemes, and RF fading parameters.

Assume that the average SNR of each bit in the FSO and RF sub-links is equal, that is, ${\bar{\gamma }}^{\mathrm{FSO}}={\bar{\gamma }}^{\mathrm{RF}}$, and the distance of the direct link is 1 km for Figure 2, Figure 3 and Figure 4. For strong turbulence, for the pointing errors parameter ξ=1 and fading parameter m=2, Figure 2 describes the relationship between the ABER performance and the SNR of the hybrid FSO/RF direct link under different modulation and detection schemes. It can be seen from Figure 2 that under the same detection scheme, the ABER performance of the hybrid FSO/RF direct link from high to low is: CBPSK, DBPSK, CBFSK, and NBFSK. Compared with frequency modulation, the ABER performance of the hybrid direct link under phase modulation is better, while the ABER performance of the hybrid direct link under coherent modulation is better than that under incoherent modulation. For example, when the heterodyne detection scheme is adopted and the SNR is 20 dB, the ABERs of the hybrid direct link with CBPSK, DBPSK, CBFSK, and NBFSK modulation are $6.159\times 10^{-6}$, $1.876\times 10^{-5}$, $3.854\times 10^{-5}$, and $1.158\times 10^{-4}$, respectively. The ABER of the hybrid FSO/RF direct link with the heterodyne detection scheme is lower than that with the direct detection scheme. For example, when the CBPSK modulation scheme is adopted and the SNR is 20 dB, the ABERs of the hybrid direct link under heterodyne detection and direct detection are $6.159\times 10^{-6}$ and $3.449\times 10^{-5}$, respectively.

With CBPSK modulation and the fading parameter m=2, Figure 3 depicts the relationship between the ABER and the SNR of the hybrid FSO/RF direct link under different turbulence intensity, detection schemes, and pointing errors. Comparing Figure 3a,b, it is easy to find that the ABER of the hybrid FSO/RF direct link with the heterodyne detection scheme is better than that of the direct detection scheme for any turbulence intensity and pointing errors. For example, for any turbulence intensity and pointing errors, the ABER of the hybrid direct link with the heterodyne detection is lower than $10^{-5}$ when the SNR is 20 dB in Figure 3a, and the ABER of the hybrid direct link with the direct detection is higher than $10^{-5}$ in Figure 3b. Although compared with only the FSO link, the performance of the hybrid FSO/RF link has been greatly improved, it can be seen from Figure 3 that the turbulence intensity and the pointing errors still have a significant impact on the ABER of the hybrid direct link. For example, in Figure 3a, with the heterodyne detection and 16 dB SNR, the ABERs of the hybrid direct link with the pointing errors parameter ξ=1 and ξ=3 under moderate turbulence are $4.001\times 10^{-5}$ and $1.445\times 10^{-5}$, respectively, and the ABERs of the hybrid direct link with the pointing errors parameter ξ=1 and ξ=3 under weak turbulence are $3.879\times 10^{-5}$ and $1.249\times 10^{-5}$, respectively.

With CBPSK modulation and ξ=1, Figure 4 shows the relationship between the ABER and the SNR of the hybrid direct link under different turbulence intensity, detection schemes, and RF fading parameters. It can be seen from Figure 3 that the RF fading parameter has a greater impact on the ABER performance of the hybrid FSO/RF direct link. When this increases, the ABER of the system increases. For example, with a 20 dB SNR and moderate turbulence, the ABERs of the hybrid direct link with m=2 and m=4 under heterodyne detection are $2.992\times 10^{-6}$ and $2.13\times 10^{-8}$, respectively, and the ABERs of the hybrid direct link with m=2 and m=4 under direct detection are $2.823\times 10^{-5}$ and $1.228\times 10^{-7}$, respectively.

**Figure 5:**
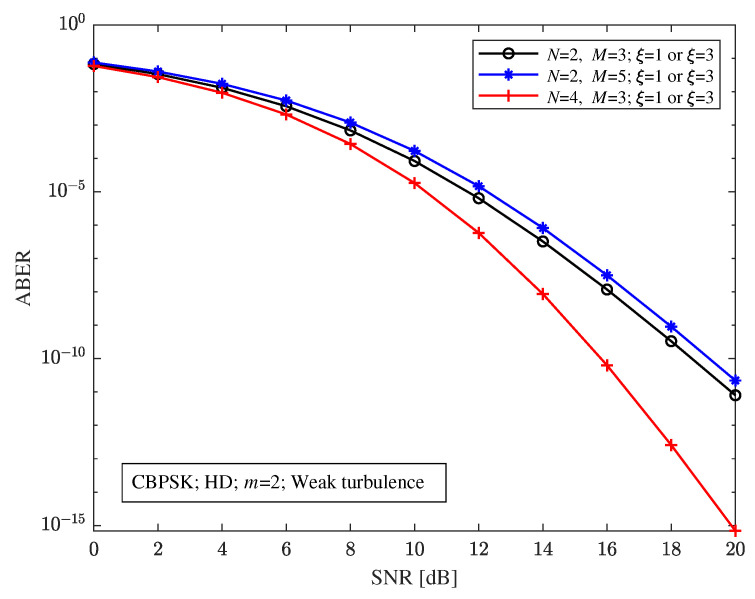
Relationship between the ABER and the SNR of the relay-aided hybrid FSO/RF cooperation system under different pointing errors and relay-aided structures.

**Figure 6:**
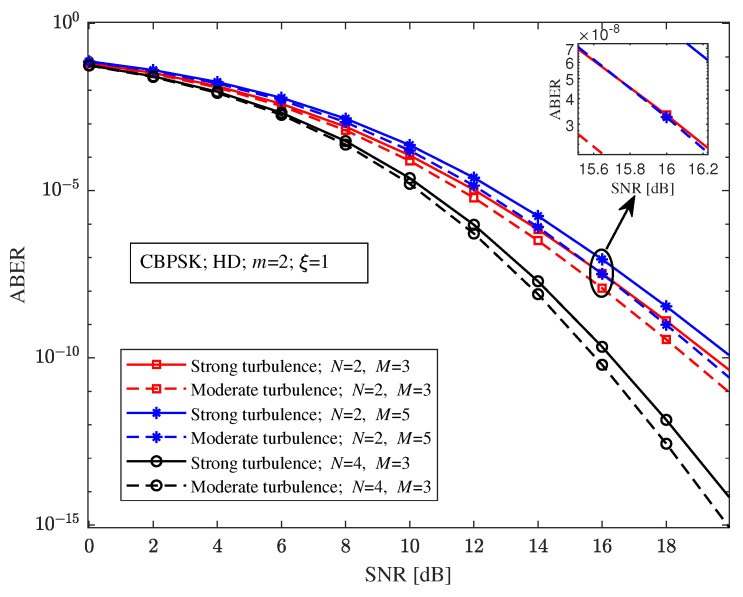
Relationship between the ABER and the SNR of the relay-aided hybrid FSO/RF system under different turbulence intensity and relay-aided structures.

**Figure 7:**
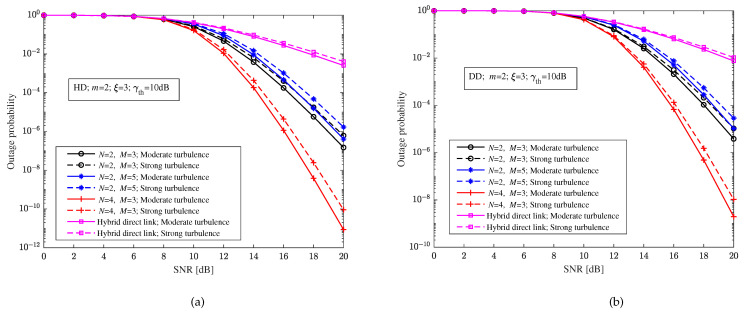
Relationship between the outage probability and the SNR of the relay-aided hybrid FSO/RF system and hybrid direct link under different turbulence intensity and relay-aided structures. (**a**) Heterodyne detection (HD). (**b**) Direct detection (DD).

**Figure 8:**
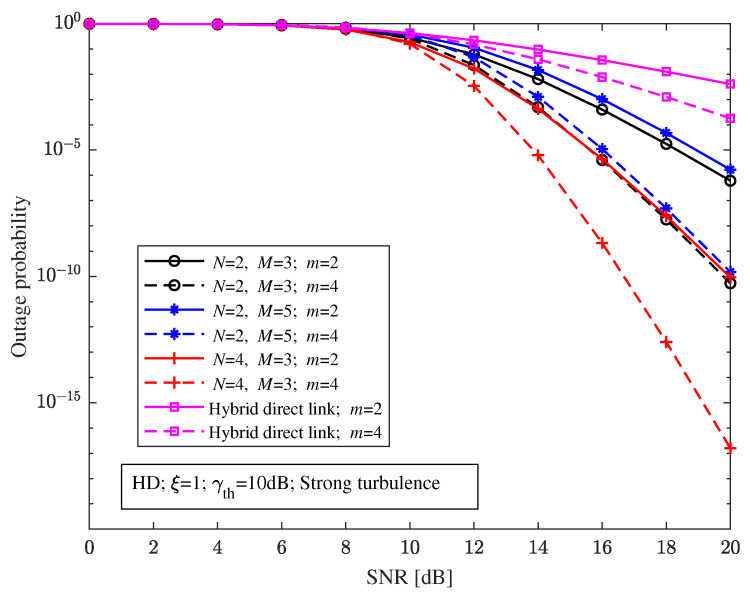
Relationship between the outage probability and the SNR of the relay-aided hybrid FSO/RF system with different structures and the hybrid direct link under different RF fading parameters.

**Figure 9:**
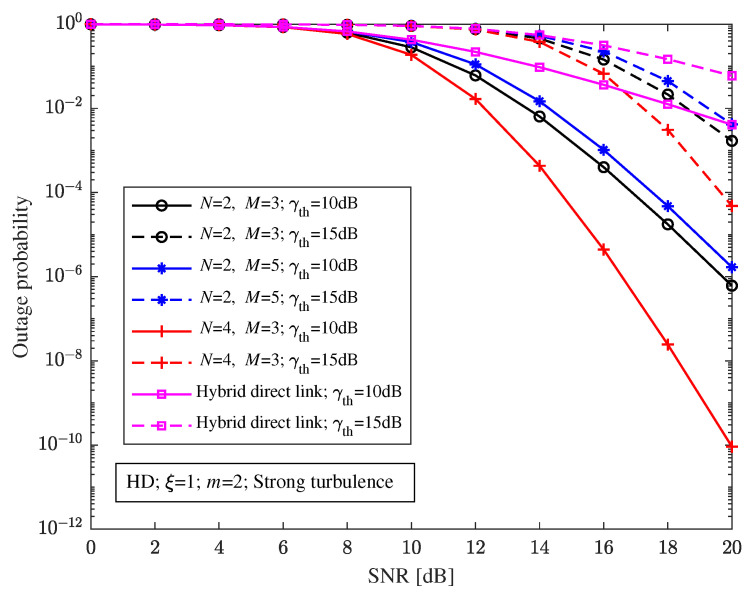
Relationship between the outage probability and the SNR of the relay-aided hybrid FSO/RF system with different structures and the hybrid direct link under different decision thresholds.

Assuming that the average SNR of each bit in the FSO and RF sub-links is equal, that is, ${\bar{\gamma }}^{\mathrm{FSO}}={\bar{\gamma }}^{\mathrm{RF}}$, and the distance of each hop and direct link is 1 km for Figure 5, Figure 6, Figure 7, Figure 8 and Figure 9, under CBPSK modulation with heterodyne detection, with the RF fading parameter m=2 and weak turbulence conditions, Figure 5 shows the relationship between the ABER and the SNR of the relay-aided hybrid FSO/RF system with different pointing errors and relay-aided structures. Figure 5 shows that the pointing errors have no impact on the ABER performance of the relay-aided hybrid system, that is, the relay-aided hybrid system can compensate for the adverse impact of pointing errors on the system. According to Figure 5, it can also be seen that different structures of the relay-aided hybrid system have different abilities to improve the ABER performance, that is, the ABER performance will be improved with the increase in the path, and will be deteriorated with the increase in the number of hops. This is because when the transmission path increases, the probability of receiving a large SNR signal at the receiving end of the system increases, resulting in the improvement of the ABER performance. When the number of hops increases, the cumulative ABER of the signal received by the receiving end of the system will increase, so the overall ABER of the system will decline. For example, when the SNR is 20 dB, the ABERs of the relay-aided hybrid systems with (N=2, M=3), (N=2, M=5), and (N=4, M=3) structures are $7.98\times 10^{-12}$, $2.206\times 10^{-11}$, and $6.925\times 10^{-16}$, respectively. Comparing Figure 4 and Figure 5, it can be further found that the relay-aided hybrid system can significantly improve the ABER performance of the system compared with the hybrid direct link.

With CBPSK modulation, heterodyne detection, the RF fading parameter m=2, and the pointing errors parameter ξ=1, Figure 6 depicts the relationship between the ABER and the SNR of the relay-aided hybrid FSO/RF system under different turbulence intensity and relay-aided structures. Figure 6 shows that the relay-aided hybrid FSO/RF system with a suitable structure can better compensate for the negative impact of turbulence and improve the ABER performance. For example, when the SNR is 20 dB, the ABERs of the relay-aided hybrid system with (N=2, M=3), (N=2, M=5), and (N=4, M=3) structures under strong turbulence are $4.088\times 10^{-11}$, $1.129\times 10^{-10}$, and $6.197\times 10^{-15}$, respectively, and the ABERs under moderate turbulence are $8.742\times 10^{-12}$, $2.417\times 10^{-11}$, and $7.723\times 10^{-16}$, respectively.

When the pointing errors parameter ξ=3, the RF fading parameter m=2, and the decision threshold $\gamma_{\mathrm{th}}=10$ dB, Figure 7 shows the relationship between the outage probability and the SNR of the relay-aided hybrid FSO/RF system and hybrid direct link with heterodyne detection and direct detection, respectively. According to Figure 7, it is easy to see that compared with the hybrid direct link, the relay-aided hybrid system can significantly improve the outage performance. In Figure 7, we can also see that a different turbulence intensity still has a certain impact on the relay-aided hybrid FSO/RF system. Different structures of the relay-aided hybrid system have different effects on the outage performance of the system, that is, the outage probability of the system will increase with the increase in hops and decrease with the increase in paths. Comparing Figure 7a,b, it can be seen that the outage probability of the relay-aided hybrid system and the hybrid direct link with heterodyne detection is better than that with direct detection.

When the pointing errors parameter ξ=1, decision threshold $\gamma_{\mathrm{th}}=10$ dB, and with strong turbulence, Figure 8 shows the relationship between the outage probability and the SNR of the relay-aided hybrid system with different structures and the hybrid direct link under different RF fading parameters. It can be seen from Figure 8 that the relay-aided hybrid system can effectively improve the outage performance compared with the hybrid direct link with different RF fading parameters *m*. The outage probability of the relay-aided hybrid FSO/RF system and the hybrid direct link will decrease with the increase in *m*.

When the pointing errors parameter ξ=1, RF fading parameter m=2, and with strong turbulence, Figure 9 shows the relationship between the outage probability and the SNR of the relay-aided hybrid system with different structures and the hybrid direct link at different decision thresholds. It can be seen from Figure 9 that the outage probability of the relay-aided hybrid system and the hybrid direct link is very sensitive to the decision threshold. Compared with the hybrid direct link, the relay-aided hybrid system with different decision thresholds can effectively improve the outage performance. The outage probability of the relay-aided hybrid system and the hybrid direct link will decrease as the decision threshold decreases.

**Figure 10:**
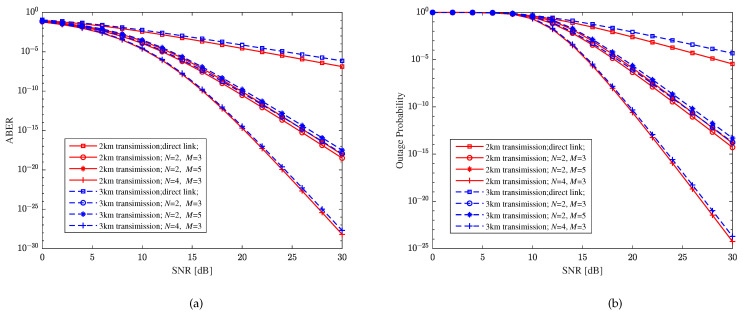
Relationship between performance metrics and the SNR of the relay-aided hybrid FSO/RF system and hybrid direct link with different direct distances. (**a**) Bit error rate (BER). (**b**) Outage probability (OP).

Assume that the average SNR of each bit in the FSO and RF sub-links is equal, that is, ${\bar{\gamma }}^{\mathrm{FSO}}={\bar{\gamma }}^{\mathrm{RF}}$, the distance of each hop is 1 km and the direct link is 2 or 3 km. With weak turbulence, CBPSK modulation, heterodyne detection, the RF fading parameter m=2, the pointing errors parameter ξ=1, and the decision threshold $\gamma_{\mathrm{th}}=10$ dB, Figure 10 shows the relationship between the performance metrics and the SNR of the relay-aided hybrid FSO/RF system under different direct distances and relay-aided structures. Similar to the previous conclusion, it can be seen from Figure 10 that as the direct distance increases, the performance of the hybrid FSO/RF system will slightly decrease, that is, the transmission distance has little impact on the performance of hybrid direct links and relay-aided hybrid systems. The hybrid direct communication system can effectively improve the reliability of the communication system. Furthermore, we can see that the relay-aided hybrid system can significantly improve the system performance, and as the number of paths increases, the system’s performance can be significantly improved, while an increase in hops slightly reduces the system’s performance.

**Figure 11:**
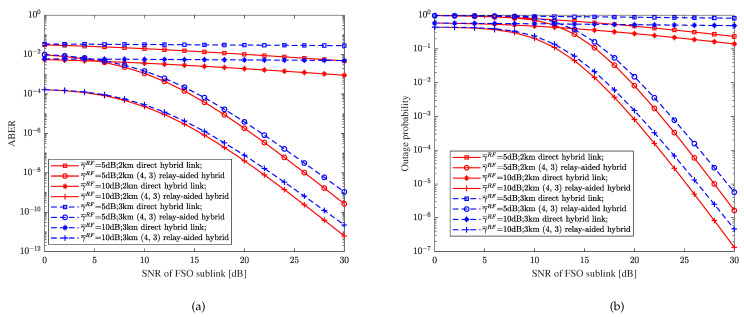
Relationship between performance metrics and the SNR of the relay-aided hybrid FSO/RF system and hybrid direct link with different direct distances and average SNR of the RF sub-link. (**a**) BER. (**b**) OP.

Assume that the average SNR of each bit in the FSO and RF sub-links is unequal, that is, ${\bar{\gamma }}^{\mathrm{FSO}}\neq {\bar{\gamma }}^{\mathrm{RF}}$, the distance of each hop is 1 km and the direct link is 2 or 3 km. With weak turbulence, CBPSK modulation, heterodyne detection, the RF fading parameter m=2, the pointing errors parameter ξ=1, and the decision threshold $\gamma_{\mathrm{th}}=10$ dB, Figure 11 shows the relationship between the performance metrics and the SNR of the relay-aided hybrid FSO/RF system under different direct distances, the SNR of the RF sub-link, and relay-aided structures. From Figure 11, it can be clearly seen that the changes in the SNR of the RF sub-links have a significant impact on the systems of hybrid direct links and relay-aided hybrid systems. Further, compared with Figure 10, we can find that the performance of the hybrid direct link and the relay-aided hybrid system with different transmission distances under the same conditions is significantly different after the SNR of the RF sub-link changes.

## 5. Conclusions

This paper studies a relay-aided hybrid FSO/RF cooperation system to further improve the performance of the FSO system. By analyzing the end-to-end instantaneous SNR of the hybrid direct link and the relay-aided hybrid system with the subcarrier modulation scheme, new mathematical expressions of the ABER and outage probability are derived. Through system simulation, the effects of different turbulence intensity, pointing errors, modulation modes, detection schemes, and RF fading parameters on the hybrid direct link and the relay-aided hybrid system are compared and analyzed.

According to the research and analysis of this paper, we can obtain some important conclusions. Compared with the hybrid direct link, the relay-aided hybrid system can further improve the performance of the system over atmospheric turbulence and pointing errors. By selecting the appropriate subcarrier modulation mode and detection scheme, the communication performance of the hybrid direct link and the relay-aided hybrid system can be improved. For example, the performance of the system with CBPSK modulation and the heterodyne detection scheme is the best in this paper. For the relay-aided hybrid FSO/RF system, the communication performance of the system can be improved by increasing the number of paths and reducing the number of hops, but the increase in the number of hops can increase the transmission distance and coverage. The influence of the RF fading parameter *m* on the hybrid direct link and the relay-aided hybrid system is obvious. The relay-aided hybrid system can greatly compensate for the deterioration of the system performance caused by pointing errors. The change in direct distance has little impact on the performance of hybrid direct links and relay-aided hybrid systems, while the change in the SNR of RF sub-links has a significant impact on their performance.

## Figures and Tables

**Table 1:** Parameters *p* and *q* for the various binary modulation schemes.

Binary Modulation Scheme	*p*	*q*
Coherent binary phase shift keying (CBPSK)	0.5	1
Differential binary phase shift keying (DBPSK)	1	1
Coherent binary frequency shift keying (CBFSK)	0.5	0.5
Non-coherent binary frequency shift keying (NBFSK)	1	0.5

## Data Availability

Not applicable.
